# Cost of Treating Pediatric Cancer at the Butaro Cancer Center of Excellence in Rwanda

**DOI:** 10.1200/JGO.17.00155

**Published:** 2018-11-15

**Authors:** Claire Neal, Christian Rusangwa, Ryan Borg, Jean Claude Mugunga, Stephanie Kennell-Heiling, Cyprien Shyirambere, Natalie Pritchett, Clemence Muhayimana, Elisephan Ntakirutimana, Neo Tapela, Paul H. Park, Lawrence N. Shulman, Tharcisse Mpunga

**Affiliations:** **Claire Neal**, University of North Carolina, Chapel Hill, NC; **Christian Rusangwa**, **Ryan Borg**, **Stephanie Kennell-Heiling**, **Cyprien Shyirambere**, **Natalie Pritchett**, **Clemence Muhayimana**, **Elisephan Ntakirutimana**, and **Paul H. Park**, Partners in Health/Inshuti Mu Buzima, Kigali; **Tharcisse Mpunga**, Ministry of Health, Butaro, Rwanda; **Jean Claude Mugunga**, Partners in Health; **Neo Tapela** and **Paul H. Park**, Brigham and Women's Hospital, Boston, MA; and **Lawrence N. Shulman**, University of Pennsylvania, Philadelphia, PA.

## Abstract

**Purpose:**

Improvements in childhood survival rates have been achieved in low- and middle- income countries that have made a commitment to improve access to cancer care. Accurate data on the costs of delivering cancer treatment in these settings will allow ministries of health and donors to accurately assess and plan for expansions of access to care. This study assessed the financial cost of treating two common pediatric cancers, nephroblastoma and Hodgkin lymphoma, at the Butaro Cancer Center of Excellence in rural Rwanda.

**Methods:**

A microcosting approach was used to calculate the per-patient cost for Hodgkin lymphoma and nephroblastoma diagnosis and treatment. Costs were analyzed retrospectively from the provider perspective for the 2014 fiscal year. The cost per patient was determined using an idealized patient receiving a full course of treatment, follow-up, and recommended social support in accordance with the national treatment protocol for each cancer.

**Results:**

The cost for a full course of treatment, follow-up, and social support was determined to be between $1,490 and $2,093 for a patient with nephroblastoma and between $1,140 and $1,793 for a pediatric patient with Hodgkin lymphoma.

**Conclusion:**

Task shifting, reduced labor costs, and locally adapted protocols contributed to significantly lower costs than those seen in middle- or high-income countries.

## INTRODUCTION

More than 80% of the approximately 200,000 children diagnosed with cancer each year live in low- and middle-income countries (LMICs).^1^ Up to 90% of childhood cancer deaths occur in low-income countries, where access to effective treatment is severely limited.^2^ Yet, many children have a high chance of cure and long-term survival (particularly for those cancers most often found in developing countries) if surgery, radiotherapy, and drugs are accessible. Research on the costs of pediatric cancer treatment in low-income countries can help inform policymakers and funders about the affordability of creating cancer treatment programs and provide information needed to advocate for greater investment in fighting this growing burden.

This study assessed the financial cost of treating two common pediatric cancers, nephroblastoma and Hodgkin lymphoma (HL), at the Butaro Cancer Center of Excellence (BCCOE), located at the Butaro District Hospital in rural northern Rwanda. We hope these findings will strengthen the argument that pediatric cancers can and should be treated in LMICs and provide evidence to support greater resource allocation.

## METHODS

### Study Setting and Population

The BCCOE is the referral center for cancer care for the country of Rwanda, offering preventive care, pathology-based diagnosis, staging, chemotherapy, referral for radiotherapy, follow-up, and palliative care, as well as psychosocial and practical support.^2-4^ Imaging and surgical services not currently available at BCCOE are provided through referral hospitals.

Between July 2013 and June 2014, 35 patients with nephroblastoma, also known as Wilms tumor, were diagnosed; this represents the most common childhood malignancy seen at the BCCOE.^2^ Seven pediatric patients with HL (and approximately 23 adult patients) were diagnosed at BCCOE between July 2013 and June 2014. Pediatric patients were defined as patients ≤ 15 years of age at the time of diagnosis.

### Cost Analysis

A microcosting approach was used to calculate the per-patient cost for HL and nephroblastoma management. Costs were analyzed retrospectively from the provider perspective for the 2014 fiscal year (July 1, 2013, to June 30, 2014). The provider perspective reflects costs incurred by the entities responsible for implementing the cancer care program. Our focus was on capturing the costs of implementing an effective cancer program and did not include patient costs, such as the out-of-pocket expenses of patients or the larger societal costs of treatment, such as lost productivity. Nor did it try to estimate what insurance coverage might be, because the national health care insurance in Rwanda does not currently cover cancer care.

The cost per patient was determined using an idealized patient receiving a full course of treatment, follow-up, and recommended social support in accordance with the national treatment protocol. The microcosting methodology was selected to allow for ministries of health and providers to better plan for the implementation of cancer programs. The sensitivity analysis demonstrates that these calculations are likely to give accurate estimates. All costs paid in Rwandan francs were converted to US dollars ($) using the conversion rate at the time of purchase, where available, and the median 2014 exchange rate of 674 Rwandan francs to 1US$ when a daily rate was not available.

#### Overhead.

An annualized total for fixed start-up costs, equipment, and supplies for the cancer center was calculated, and a proportion was assigned based on the total number of patients with cancer. All annual overhead costs for the oncology program (including security, administration, sewage, electricity, supplies and consumables, transport for blood products, landscaping, and maintenance) were assigned to each patient with cancer treated on the basis of patient volume and time on the ward. During the study period, most chemotherapy was administered as an in-patient treatment. Time on the ward was calculated assuming a total of 40 days for HL and 52 days for nephroblastoma, including both treatment and follow-up. The estimates are consistent with the average hospitalization time for a full course of treatment and one adverse event.

#### Chemotherapy and supportive care medications.

All chemotherapy and supportive care drugs were enumerated following the established treatment protocol for each disease in Rwanda. Chemotherapy costs for nephroblastoma were calculated assuming a child with metastatic disease stage and a body surface area of 0.6 m^2^ and weight of 15 kg. Chemotherapy costs for HL were calculated assuming a child with stage III HL, a body surface area of 1 m^2^ and a weight of 30 kg. Drug prices were calculated based on prices paid by the Partners in Health procurement team at the time of treatment and vial usage. The Partners in Health procurement team is able to negotiate prices across their delivery sites, resulting in economies of scale. Oncology drugs are exempt from taxation in the country. On the basis of vial usage, the methodology accounts for drug waste and leads to results that are both more accurate and more conservative.

#### Computed tomography scans and surgery.

Computed tomography (CT) scans and surgery were conducted off site at a referral hospital in Kigali. The 2012 Rwandan National Reference for Rates and Hospital Centres^5^ was used to determine the full cost of appropriate CT scans and surgery per patient with nephroblastoma. CT scans are currently not part of the protocol for pediatric HL and were therefore not included.

#### Pathology.

The majority of pathology costs are incorporated in labor and overhead calculations. Additional costs for the program included items such as shipment of samples to Boston, training and travel costs for pathology laboratory technicians, customs and duty charges for reagents, and consumables related to the pathology laboratory. The average cost per patient for these expenses was allocated on the basis of all new diagnoses at BCCOE during the study period.

#### Radiotherapy.

Radiotherapy is not available in Rwanda; patients needing this treatment must be referred out of the country. Radiotherapy is used in limited situations where its inclusion in the treatment plan is deemed as essential for cure. The average cost of radiotherapy per patient was adjusted to reflect the low volume of patients receiving this treatment for nephroblastoma. If residual disease exists after chemotherapy, in rare cases, a patient with HL can be sent for radiotherapy. Given the rarity of this occurrence, this cost was not included in our analysis.

#### Blood products.

All blood products are provided free of charge to hospitals through the National Center for Blood Transfusion Division of the Rwanda Biomedical Center. The cost given by the National Center for Blood Transfusion for each RBC unit is $88. Provider interviews were used to determine an average use of 5 RBC units per patient with nephroblastoma and 7 RBC units per patient with HL over the treatment cycle.

#### Transportation costs.

BCCOE is located in a remote district of Rwanda 3 hours from the capital, and funding for transportation is provided to each patient in need to ensure that patients can access treatment. The median round trip cost of transportation was combined with the average number of trips for a full course of treatment and follow-up to determine the full cost of supporting transportation for treatment per patient.

#### Labor costs.

We identified all relevant hospital staff involved in the treatment of pediatric patients with cancer, including doctors, nurses, social workers, cashiers, laboratory technicians, intake coordinators, and pharmacy staff. We then interviewed physicians, nurses, technicians, and administrative staff to determine the major activities performed in treating each type of cancer and created process maps to estimate the amount of time each type of provider spent on each activity with each patient. We adjusted staff salaries by the estimated total number of paid hours worked each year to determine a cost per hour for each provider. The provider cost per hour was allocated to the relevant patient care activities to create a total staff cost per patient. The analysis assumes one adverse event per patient on the basis of provider interviews of the typical experience and treatment decisions made for these specific cancers. Given the short duration of treatment, we did not apply a discount rate. We conducted a one-way sensitivity analysis to determine the parameters most likely to affect the cost per patient.

## RESULTS

### Nephroblastoma

The total cost of management for a patient with metastatic nephroblastoma was $2,093 (Table 1). Labor and blood products were the primary cost drivers, accounting for 20% and 21% of costs, respectively. Chemotherapy (17%) as well as CT scans and surgeries (18%) also accounted for large proportions of the overall cost. Chemotherapy costs per patient are listed in Table 2.

**Table 1:**
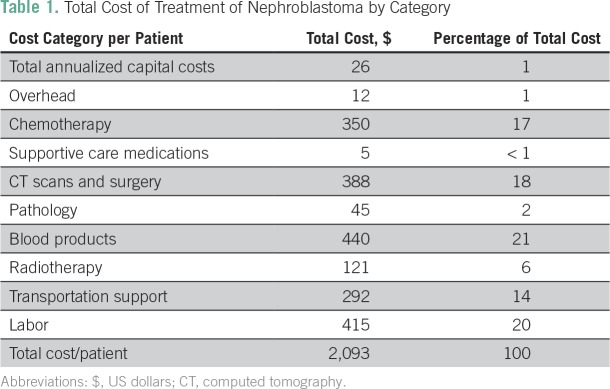
Total Cost of Treatment of Nephroblastoma by Category

**Table 2:**
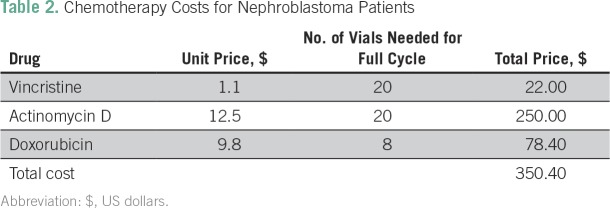
Chemotherapy Costs for Nephroblastoma Patients

All qualifying patients in need receive transportation support to ensure that they are able to reach treatment. As a result, transportation costs represent a significant proportion of the total cost for the provider (14%); however, it is important to note this is based on the assumption that a patient will require support for every trip during a full course of treatment and follow-up. Radiotherapy represents only 6% of the total cost because of the small proportion of patients receiving this treatment option. Capital costs, overhead, and supportive care medications are all minimal costs at the patient level.

We conducted a one-way sensitivity analysis to determine how changes in our assumptions might affect the analysis and to identify the parameters most likely to affect the cost per patient (Table 3). We varied the primary cost components most likely to change by 20% to reflect expected changes in labor, chemotherapy prices, blood products, and CT scans. We varied the number of hospital days by 50% to reflect either an additional adverse event or the expected changes that would occur when shifting to an outpatient (or infusion center) model. None of the parameters had a significant (greater than 5%) effect on the overall cost of care.

**Table 3:**
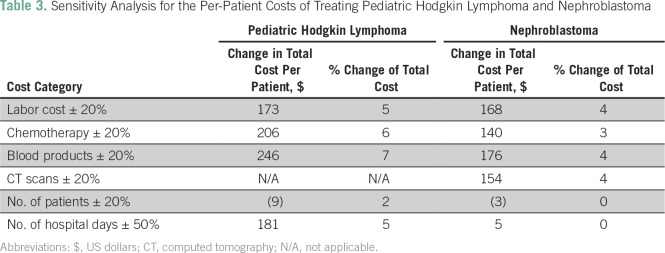
Sensitivity Analysis for the Per-Patient Costs of Treating Pediatric Hodgkin Lymphoma and Nephroblastoma

The current cost per patient diagnosed with nephroblastoma is based on a patient with metastasis completing a full cycle of treatment and follow-up. Of course, not all patients will present at the same stage, have the same disease progression, or complete treatment and/or follow-up. If one assumes that outcomes will continue to be similar to the initial outcomes seen in Rwanda for patients with Wilms tumor treated at BCCOE^6^ (including approximately 39% of patients completing treatment and all follow-up), the adjusted average cost was $1,490 per patient. The cost of $2,093 represents the idealized cost of treating a patient with nephroblastoma, whereas $1,490 was the per-patient cost adjusted for expected survival and loss to follow-up outcomes at BCCOE.

### Pediatric HL

The total cost for a full course of treatment, follow-up, and recommended social support for a patient with HL is estimated to be $1,793 (Table 4). Blood products and chemotherapy are the primary cost drivers, accounting for 34% and 29% of the total cost, respectively. A full course of chemotherapy costs $514.20 (Table 5). Labor is also a significant cost driver, at 24%. Blood products were the parameter most sensitive to change in the one-way sensitivity analysis, at 7% (Table 3), followed by chemotherapy, at 6%, with days on the ward, number of patients, and labor costs all reflecting no more than a 5% change.

**Table 4:**
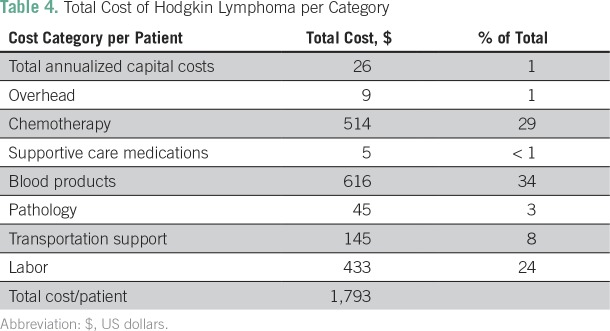
Total Cost of Hodgkin Lymphoma per Category

**Table 5:**
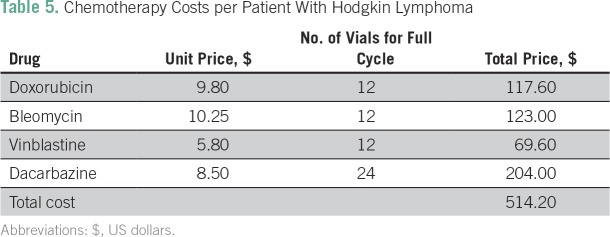
Chemotherapy Costs per Patient With Hodgkin Lymphoma

Assuming we can expect similar completion rates to continue at BCCOE (including 68% of patients completing both treatment and follow-up),^7^ the total cost of treatment adjusted for expected survival and loss to follow-up was estimated to be $1,140 per patient, representing a reduction of $652 per patient.

## DISCUSSION

The cost for a full course of treatment and follow-up was determined to be between $1,490 and $2,093 for a patient with nephroblastoma and between $1,140 and $1,793 for a pediatric patient with HL. The cost of pediatric cancer treatment in Rwanda is significantly lower than that seen in high-income countries, often estimated to be between $40,000 and $100,000, depending on cancer type.^8^ Accurate assessments of the costs for low-income countries can help both donors and governments make informed investment decisions with regard to cancer treatment facilities. These assessments can affect political will to dedicate resources, even when costs are high relative to other potential investments.^9^

A study conducted in the Kigali University Teaching Hospital in Rwanda found that treatment costs for nephroblastoma were consistent with the findings of this study, with costs between $1,831 for early disease and $2,418 for advanced disease.^6^ Given the different methodologies and the low number of patients in each study, the consistent results between the two studies helps to bolster the case for the accuracy of these costs. A study conducted in two South African hospitals found that the total cost of diagnosing, staging, and treating a child with HL was $6,647.^10^ Our analysis found a much lower cost per patient. CT scans and radiotherapy were included as standard components of management in the South African study. This accounted for a significant difference in cost because radiotherapy and CT scans were not standard components of care in the Rwandan analysis. However, if CT scans were more widely available and radiotherapy could be provided in the country, they could become standard components of care at BCCOE as well.

Drug prices also reflect a large portion of the difference; in Rwanda, the course of chemotherapy is approximately one fifth of the cost outlined in the South African study. Despite the lower cost of chemotherapy in Rwanda and the use of generic drugs, chemotherapy still represents a significant cost. As cancer treatment becomes more widely available, collective bargaining could be leveraged to further reduce costs. The example of HIV/AIDS medications is particularly instructive, because concerted advocacy efforts and bulk pricing reduced medication costs in low-income countries by as much as 90% from the price when first introduced in high-income countries.^9^ Improving cost transparency would also help with negotiations to adjust costs appropriately for different settings.

A number of factors contribute to the ability of BCCOE to provide quality and effective cancer care at lower costs. As with many developing countries, the cost of labor for the cancer center is significantly less expensive than in more economically developed countries. Labor costs for the entire cancer center (including all positions, from doctors, nurses, and nutritionists to custodians and cashiers) is less than the average annual salary of $246,526 for one oncologist in the United States.^11^ Rwanda uses a task-shifting model to allow generalists (pediatricians, internists, and general practitioners) to provide care with structured support from volunteer US-based oncologists, further reducing costs.^2^ Locally adapted protocols that minimize toxicity and length of stay in the hospital (and are therefore less expensive) have been shown to cure a significant number of children when applied with appropriate social support in low-resource settings.^12,13^

The analysis is limited in that the cost per patient was determined using an idealized patient receiving a full course of treatment and follow-up, in accordance with the current Rwandan protocol for treatment of each cancer. Often, the complexity, toxicity, and length of treatment combined with the availability of resources can profoundly affect the cost of care stemming from these protocols. Protocols may be further adapted to the local context over time. As the recommended protocols are further refined, we can expect to see corresponding changes in the cost of treatment. In addition, limitations of the program include a high volume of patients for limited bed space and staff time. As more hospital beds and paid staff are made available, the cost per patient may increase as each patient receives a greater share of available resources.

A number of data points were restricted or unavailable. The estimates of the costs of CT scans and surgery in our analysis are based on the prices charged by the relevant referral hospitals. We did not have access to the data necessary to perform a full and accurate accounting of the costs of these procedures on the basis of actual resource consumption. Because a relatively small percentage of patients receive these services, this cost analysis has limited sensitivity (4%). However, a more accurate cost of these procedures would further refine our estimates. Labor costs produced from provider interviews are subject to recall bias. Multiple providers’ opinions were sought to minimize this bias as much as possible. In addition, the BCCOE relied on volunteer labor, including volunteer doctors, nurses, and pathologists. The cost per patient assumes all providers are paid staff and does not take into account free labor. Therefore, this methodology likely overstates the true cost per patient.

The one-way sensitivity analysis (Table 3) revealed that none of the parameters had a significant impact on the overall cost of care. The parameters most sensitive to change include the cost of labor, blood products, and CT scans. For both cancers, increasing or decreasing the cost of chemotherapy by 20% resulted in a less than 6% change to the overall cost per patient. A change of 50% in radiotherapy costs produced only a 3% change in the overall cost per patient. This is the result of the small number of patients (4%) currently receiving radiotherapy; if the protocol was adjusted and a large number of patients received radiotherapy, we would expect significant changes in this cost component. We varied the expected change in the number of days by 50% to reflect either the significant reductions in hospital days expected when moving to an outpatient infusion center or the increase in the number of days if a patient were to have many additional adverse events

In conclusion, the majority of new pediatric cancers are now occurring in LMICs, and these countries are often the least equipped to respond to the growing burden.^14^ The cost of treatment provides a strong call to action for the global health community to support the establishment of new cancer programs, particularly considering that the long-term survival rates for children with cancer in high-income countries exceed 80%. Innovative approaches, such as those used at BCCOE, which combine a strong commitment to cancer care, adaptation of protocols to the local setting, task shifting, and collaborative partnerships, have great potential to produce affordable, quality cancer care. This study demonstrates that an investment of less than $2,100 per patient can save children’s lives and protect families from the hardship and heartache of losing a child to cancer.
